# Light meson physics at BESIII

**DOI:** 10.1093/nsr/nwab052

**Published:** 2021-03-31

**Authors:** Shuang-shi Fang

**Affiliations:** Institute of High Energy Physics, Chinese Academy of Sciences, Beijing 100049, China; University of Chinese Academy of Sciences, Beijing 100049, China

**Keywords:** light meson decays, charmonium decays, *e* ^+^ *e* ^−^ annihilation, BESIII detector

## Abstract

Studies of light meson decays are important tools to perform precision tests of the effective field theories, determine transition form factors and test fundamental symmetries. With very high statistics data samples, the Beijing Spectrometer III (BESIII) experiment provides a unique laboratory for light meson studies and is contributing significantly to a variety of these investigations. A brief review of recent progress in light meson decay studied at the BESIII experiment, including detailed studies of common decay dynamics, searches for rare/forbidden decays and new particles, is presented. Finally, together with descriptions of different experimental techniques, prospects for future studies of light mesons are discussed in some detail.

## Teaser

Studies of light mesons continue to provide opportunities for exploring a variety of physics at low energy scales. The BESIII experiment will bring the study of light meson decays into a precision era and make significant contributions to understanding of hadron physics in the non-perturbative regime.

## INTRODUCTION

The discovery of light mesons and detailed studies of their decays have played crucial roles in the development of our understanding of elementary particle physics. In the case of weak interactions, important insights were gained from kaon and pion decays, such as the observation of *CP* violation and validation of the V-A structure of the theory. In addition, the discovery of strangeness inspired the SU(3) flavor symmetry, which, in turn, gave the birth to the quark model picture of the underlying structure of observed particles. To date, about seven decades since the discovery of the first light mesons (the pion and kaon), studies of light meson decays continue to provide opportunities for a variety of physics at low-energy scales, including precision tests of effective field theories, investigations of the quark structure of the light mesons, tests of the fundamental symmetries and searches for new particles.

The Beijing Spectrometer III (BESIII) experiment [1] collected the world’s largest samples of 1.3 × 10^9^*J*/ψ events [2] and 4.5 × 10^8^ ψ(3686) events [3] produced directly from *e*^+^*e* annihilation in 2009 and 2012. Because of the high production rates of light mesons in the charmonium decays, these data, in combination with the excellent performance of the detector, offer unprecedented opportunities to explore the light meson decays. Moreover, the BESIII data sample of *e*^+^*e*^−^ annihilation events at energies between 2.0 and 3.08 GeV with an integrated luminosity of  650 pb^−1^ allows for explorations of properties of the light vector mesons, in particular the vector strangeonium states.

## PRECISION TESTS OF QCD AT LOW ENERGIES

At high energies, QCD serves as a reliable and useful theory, whereas at low energies non–perturbative QCD calculations are usually performed by an effective field theory called chiral perturbation theory (ChPT). High-quality and precise measurements of low-energy hadronic processes are necessary in order to verify the systematic ChPT expansion. Thus, studies of light meson decays are important guides to our understanding of how QCD works in the non-perturbative regime.

### Light quark mass ratios in **η/η^′^ →** 3π decays

The decay of the η meson into 3π violates isospin symmetry, which is related to the difference of light quark masses, *m*_*u*_ ≠ *m*_*d*_. Therefore, the decay of η → 3π offers a unique way to determine the quark mass ratio $Q^2\equiv (m_s^2-{\hat{m}}^2)/(m_d^2-m_u^2)$ (where ${\hat{m}} = \frac{1}{2}(m_d + m_u)$). Extensive theoretical studies have been performed within the framework of combined ChPT and dispersion theory [4–9].

In addition to the recent results from the WASA-at-COSY [10] and KLOE-2 [11] experiments, BESIII reported a Dalitz plot analysis of η → 3π decays [12]. The measured matrix elements are in agreement with the most precise KLOE-2 determination and theoretical predictions. Taking experimental results as input, two dedicated analyses presented the results *Q* = 22.0 ± 0.7 [13] and *Q* = 21.6 ± 1.1 [14]. In the near future, the study of η → π^+^π^−^π^0^ and η → π^0^π^0^π^0^ decays at BESIII will provide an independent check of these results by directly fitting to differing theoretical models.

Historically, the η^′^ → π^+^π^−^π^0^ decay was considered to proceed via π^0^−η mixing [15], which offered the possibility of comparable strength *u* − *d* quark mass difference from the branching fraction ratio of $r={{{\cal B}}(\eta ^{\prime }\rightarrow \pi \pi \pi )}/{\mathcal {B}(\eta ^{\prime }\rightarrow \pi \pi \eta )}$. However, it was subsequently argued that the decay amplitudes are strongly affected by the intermediate resonances [16], e.g. the *P*-wave contribution from η^′^ → ρπ, and, thus, the *u* − *d* quark mass difference could not be extracted in such a simple way.

**Figure 1.**
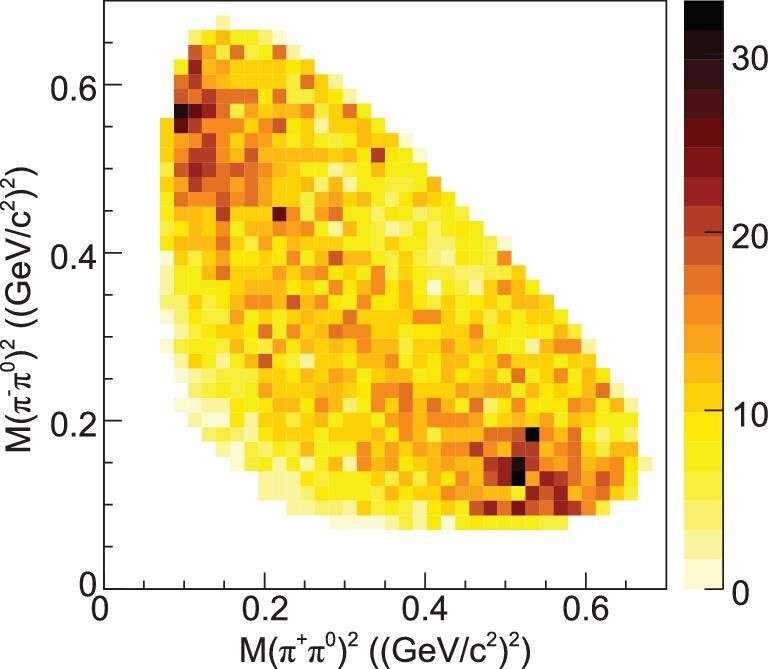
Dalitz plot of *M*^2^(π^+^π^0^) versus *M*^2^(π^−^π^0^) for the η^′^ → π^+^π^−^π^0^ decay, where the two clear clusters correspond to the η^′^ → ρ^∓^π^±^ decay [17].

In addition to the first observation of η^′^ → ρ^±^π^∓^ (Fig. 1) by BESIII [17], the resonant π–π*S* wave, interpreted as the broad *f*_0_(500), is also expected to play an essential role in η^′^ → π^+^π^−^π^0^ decays. The contribution of *f*_0_(500) provides a reasonable explanation for the negative slope parameter of the Dalitz plot of η^′^ → π^0^π^0^π^0^ [12]. Because of limited statistics, it has been impossible to differentiate between *S* and *D* waves; larger event samples are crucial for carrying out amplitude analyses of these processes. Several theory groups have expressed interest in describing the decay using a dispersive approach. These improved theoretical studies along with more precise experimental measurements of η/η^′^ → 3π decays from a variety of experiments are expected to improve the accuracy of the quark mass ratio.

### Cusp effect in **η^′^ → π^0^π^0^η** decays

In addition to the precision tests of effective theoretical models, common to all η^′^ → ππη decays, the neutral decay η^′^ → π^0^π^0^η also allows us to examine the cusp effect, i.e. an abrupt change in the π^0^π^0^ invariant mass distribution as it crosses the 2$m_{\pi ^+}$ threshold. An accurate measurement of the cusp effect may enable a determination of the *S*-wave pion–pion scattering lengths to high precision.

For η^′^ → π^+^π^−^η, BESIII results [18] are not particularly consistent with theoretical predictions based on the chiral unitary approach [19]. The discrepancies show up as about 4 SD on some of the parameters that are used to describe the Dalitz plot distribution. In the case of η^′^ → π^0^π^0^η, the results are in general consistent with theoretical predictions within the uncertainties and the latest results reported by the A2 experiment [20]. Because of the limited statistics, the present results are not precise enough to firmly establish isospin violation and additional effects, e.g. radiative corrections [21], and the π^+^/π^0^ mass difference should be considered in future experimental and theoretical studies.

A BESIII search for the cusp in η^′^ → ηπ^0^π^0^ performed by inspecting the π^0^π^0^ mass spectrum close to the π^+^π^−^ mass threshold [18] revealed no statistically significant effect. From an experimental perspective, the available high statistics of 10 billion *J*/ψ events at the BESIII experiment is expected to increase the η^′^ decay event sample by nearly an order of magnitude. These additional data coupled with the incorporation of recent dispersive theoretical analyses [22] make investigation of the cusp effect in this channel very promising.

### Box anomaly in the **η/η^′^ → γπ^+^π^−^** decay

In the vector meson dominance (VMD) model, the main contribution to the decay η^′^ → γπ^+^π^−^ comes from η^′^ → γρ. However, a significant deviation in the dipion distribution between the theoretical predictions and data is observed, and this may be attributable to the Wess-Zumino-Witten box anomaly [23,24]. Previous measurements [25–30] sometimes give opposite conclusions on the presence of the box anomaly term.

**Figure 2.**
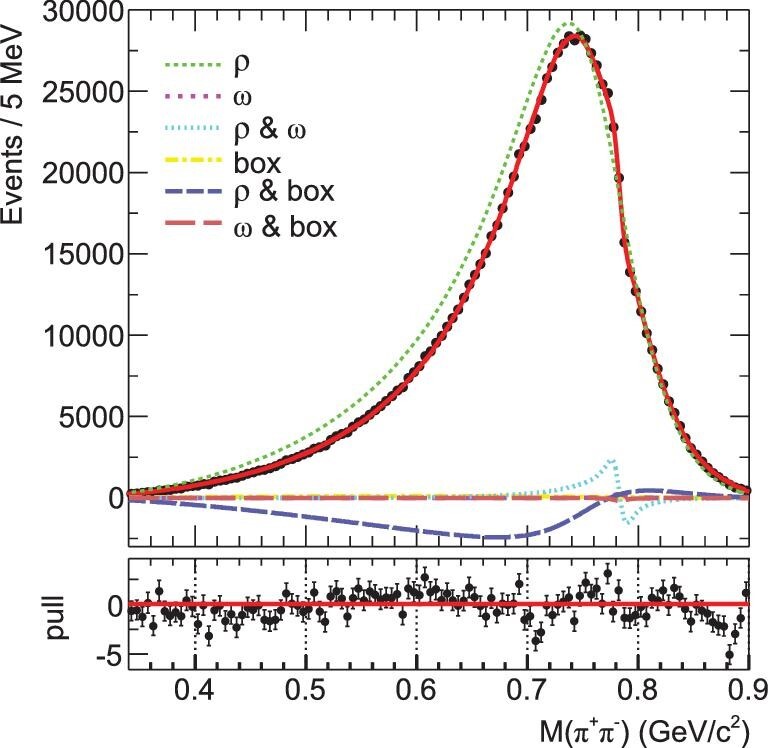
The results of model-dependent fits to *M*(π^+^π^−^) with a ρ^0^-ω box anomaly [31].

Recently, a precision BESIII study of η^′^ → γπ^+^π^−^ [31] found, for the first time, that a fit that only included the components of ρ and ω and their interference failed to describe the data; a significant additional contribution, either the box anomaly or a ρ(1450) component, is found to be necessary, as indicated in Fig. 2, to provide a good description of the data. In this case, the influence of the box anomaly phenomenon, i.e. the presence of a well-defined contact term, still requires a definite and unambiguous demonstration.

The large and clean η/η^′^ sample produced in *J*/ψ decays at the BESIII experiment is expected to promote the study of η/η^′^ → γπ^+^π^−^ to an unprecedented precision era. Along with a recently proposed model-independent approach [32], a combined analysis of η/η^′^ → γπ^+^π^−^ may present a consistent picture for the dynamics of these two decays.

### Test of higher-order ChPT with **η/η^′^ → γγπ^0^ and η^′^ → γγη** decays

The η/η^′^ → γγπ^0^ decays are of particular interest for tests of ChPT at the two-loop level. Since light vector mesons play a critical role in these models, the dynamical role of the vector mesons has to be systematically included in the context of either the VMD or Nambu-Jona-Lasinio model to reach a deeper understanding of these decays.

The η → γγπ^0^ decay has been measured in many experiments [33]. Of interest is that the branching fraction of η → γγπ^0^, (8.4 ± 2.7 ± 1.4) × 10^−5^ [34], as reported by KLOE is approximately a factor of 3 lower than that from the A2 experiment [35]. Experimentally, both the η^′^ → γγπ^0^ [36] and η^′^ → γγη [37] decays were studied at BESIII. The measured branching fractions are in agreement with a recent theoretical calculation based on the linear sigma model with VMD couplings [38]. It was also found that the di-photon invariant mass dependence of the partial decay widths differs in shape from the predictions of different theoretical models [38]. Thus, a precision measurement of the di-photon mass spectrum would be a more sensitive tool for testing the reliability of theoretical calculations than just measurements of the branching fraction. In this case, an updated measurement for these double radiative decays using the full *J*/ψ sample at the BESIII experiment will provide an opportunity to have a combined analysis that will distinguish between different theoretical models.

### Transition form factors of light mesons

The η/η^′^ → γ*l*^+^*l*^−^(*l* = *e*, μ) Dalitz decays, where the lepton pair is formed by internal conversion of an intermediate virtual photon and the decay rates are modified by the electromagnetic structure arising at the vertex of the transition, are of special interest. Deviations of measured quantity from their QED predictions are usually described in terms of a timelike transition form factor, which, in addition of being an important probe into the meson’s structure [39], has an important role in the evaluation of the hadronic light-by-light contribution to the muon anomalous magnetic moment (see [40] for details).

**Figure 3.**
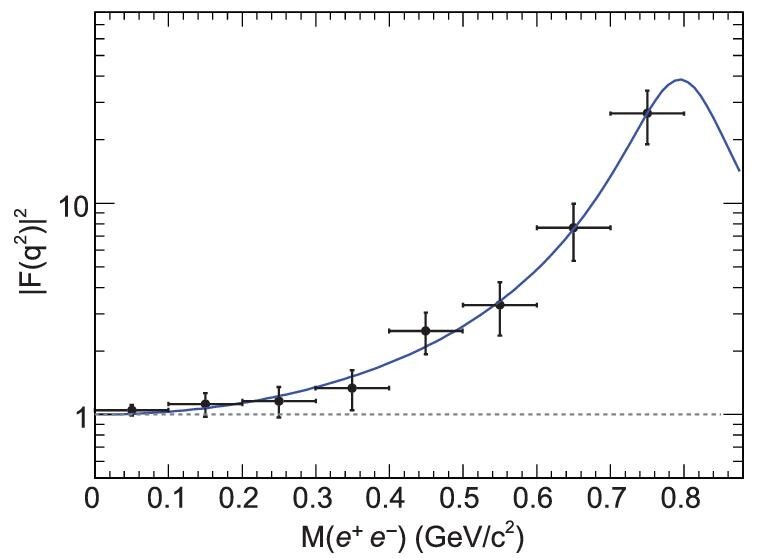
Fit to the single-pole form factor |*F* (*q*^2^)|^2^, where *q*^2^ is the square of the *e*^+^*e*^−^ invariant mass [43].

In contrast to the SND and WASA experimental studies of η → γ*l*^+^*l*^−^ [41,42], BESIII has a unique advantage in the study of Dalitz decays of both η and η^′^ due to their high production rate in *J*/ψ radiative and hadronic decays. BESIII reported the first measurement of the *e*^+^*e*^−^ invariant-mass distribution for η^′^ → γ*e*^+^*e*^−^ [43]. It was found that the single-pole parameterization provides a good description of data, as illustrated in Fig. 3. The corresponding slope parameter, $b_{\eta ^{\prime }}=1.56\pm 0.19$ GeV^−2^, is in agreement with the predictions from different theoretical models [44–47] and a 1979 previous measurement of η^′^ → γμ^+^μ^−^ [48].

The η/η^′^ → *l*^+^*l*^−^*l*^+^*l*^−^ decays address decays via two off-shell photons and indicate whether double vector meson dominance is realized in nature. To date, only the decay η → *e*^+^*e*^−^*e*^+^*e*^−^ was observed by the KLOE experiment [49]. The corresponding form factor has neither been measured in the timelike nor the spacelike region. In accordance with the theoretical investigation in [50] of the predicted decay rates of η^′^ → *e*^+^*e*^−^*e*^+^*e*^−^ of the order of 10^−4^, hundreds of events are expected to be observed by the BESIII experiment and significant progress could be made to test the latest theoretical prediction of 2.1 × 10^−6^ [51] based on a data-driven approach.

In addition, using the data sample collected at a center-of-mass energy of 3.773 GeV by the BESIII experiment, studies [52] show that the measurements of the spacelike transition form factors in the decay *e*^+^*e*^−^ → *e*^+^*e*^−^π^0^(η, η^′^) via γγ interactions in the range of the transfer momentum *Q*^2^ within [0.3, 10] GeV/c^2^ are feasible. It is worth mentioning that more data samples at 3.773 GeV and higher are planned for the BESIII experiment. They will be useful for the spacelike transition form factor measurements that are complementary to the data from other experiments and uniquely cover the *Q*^2^ range that is relevant to the hadronic light-by-light correction for the evaluation of the muon anomaly moment.

### Cross channel effect in **ω → π^+^π^−^π^0^** decays

The decay ω → π^+^π^−^π^0^ is usually employed to investigate the ω decay mechanism by comparing a high-statistics Dalitz plot density distribution with theoretical predictions. In the dispersive theoretical framework [53,54], the Dalitz plot distribution and integrated decay width are sensitive to the so-called crossed-channel effect [54]. However, prior to BESIII, no experimental ω → π^+^π^−^π^0^ data of sufficient precision were available to compare with the predictions.

Because of the high production rate of ω in *J*/ψ hadronic decays, BESIII was able to perform a precision Dalitz plot analysis with a sample of 2.6 × 10^5^ ω → π^+^π^−^π^0^ events [55], which is about 6 times larger than the samples in the previous work [56] by WASA-at-COSY. It was found that the Dalitz plot distribution of data significantly differs from the pure *P*-wave phase space, and additional contributions from resonances and/or final-state interactions are necessary. However, with the present statistics, the experimental results are consistent with the theoretical predictions without the need for incorporating crossed-channel effects, which may indicate that the crossed-channel effect contributions are overestimated in the dispersive calculations. Thus, investigation of this decay dynamics with higher precision by analyzing the full *J*/ψ data sample is needed to clarify this issue.

## QUARK STRUCTURE OF LIGHT SCALAR MESONS

The nature of the light scalar mesons *f*_0_(500), $K^*_0(800)$, *a*_0_(980) and *f*_0_(980) has been a controversial issue for several decades. Taking into account the observations in heavy meson decays, the existence of these scalar mesons is not controversial, though $K^*_0(800)$ is still qualified as ‘needs confirmation’ in the Particle Data Group listings [33]. However, the properties of these scalar mesons cannot be understood as simple $q\bar{q}$ mesons, and non-$q\bar{q}$ interpretations of the light scalar nonet are supported by a variety of theoretical approaches [57–60].

Compared to scattering experiments, *J*/ψ decays provide a clean laboratory to explore these scalar states. At BESIII, a series of amplitude analyses were performed to study scalar mesons decay into pseudoscalar meson pairs ππ, $K\bar{K}$ and π*K* in *J*/ψ decays [61–63] that established the existence of the *f*_0_(500) and K^*^(800).

**Figure 4.**
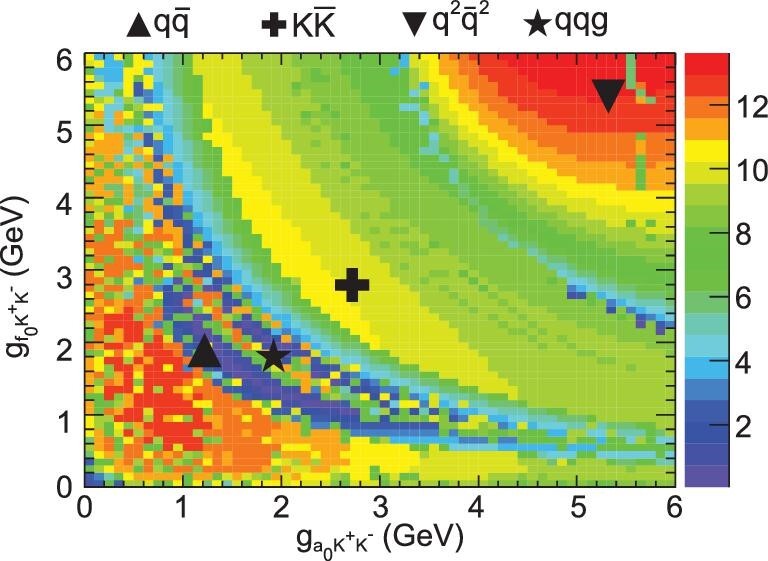
The statistical significance of the signal scanned in the two-dimensional space of $g_{a_{0}K^{+}K^{-}}$ and $g_{f_{0}K^{+}K^{-}}$ [64], where the markers indicate predictions from various illustrative theoretical models. The regions with higher statistical significance indicate larger probability for the emergence of the two coupling constants.

At BESIII, the *a*_0_(980) − *f*_0_(980) mixing effect, an essential approach for probing their nature, was observed for the first time in studies of *J*/ψ → φηπ^0^ and χ_*c*1_ → π^0^π^+^π^−^ decays [64]. The anomalous shape of *a*_0_(980) and the very narrow *f*_0_(980) peak produced by the mixing effect was clearly seen in the ηπ^0^ and π^+^π^−^ mass spectra. The significance of the mixing effect was then investigated as a function of the two coupling constants $g_{a_{0}K^{+}K^{-}}$ and $g_{f_{0}K^{+}K^{-}}$, and compared with different models for the mesons’ substructure, as shown in Fig. 4. The results favor the tetraquark model, although other possibilities still cannot be completely ruled out.

In addition to their production via charmonium decays, other processes can also be used to explore the properties of scalar mesons at BESIII, including light meson and charm meson decays. Examples are the prominent *f*_0_(500) contribution in η^′^ → 3π decays [17], and the evident effects of *a*_0_(980) − *f*_0_(980) mixing in an amplitude analysis of $D_s^+\rightarrow \pi ^+ \pi ^0\eta$ [65]. Scalar mesons copiously produced in these decays are further evidence that the BESIII experiment is a unique facility for understanding the controversial nature of these particles.

## PRECISION TESTS OF FUNDAMENTAL SYMMETRIES

The η and η^′^ mesons are eigenstates of *P*, *C* and *CP* whose strong and electromagnetic decays are either anomalous or forbidden to lowest order by *P*, *C*, *CP* and angular momentum conservation. Therefore, their decays provide a unique laboratory for testing the fundamental symmetries in flavor-conserving processes, which was extensively reviewed in [66].

A straightforward way to test these symmetries is to search for *P*- and *CP*-violating η/η^′^ decays into two pions. In the standard model (SM), the branching fractions for these modes are very tiny [67], but they may be enhanced by *CP* violation in the extended Higgs sector of the electroweak theory [68]. The high production rate for η^′^ mesons in *J*/ψ decays enabled BESIII to report the best experimental limit to date, 4.5 × 10^−4^, for $\mathcal {B}(\eta ^\prime \rightarrow \pi ^0\pi ^0)$ [69] at the 90% confidence level. More recently, BESIII made a search for the rare decay of η^′^ → 4π^0^ and reported the branching upper limit, ${\cal {B}}(\eta ^\prime \rightarrow 4\pi ^0)<3.8 \times 10^{-5}$ at the 90% confidence level, for the first time [70].

Another interesting signal for possible *CP*-violating mechanisms would be an asymmetry in the angle between the π^+^π^−^ and *e*^+^*e*^−^ planes in the η/η^′^ rest frame, where the asymmetry would be caused by the interference between the usual *CP* allowed magnetic transition (driven by the chiral anomaly) and a *CP*-violating flavor-conserving electric dipole operator [71]. The experimental bound on this asymmetry for η → π^+^π^−^*e*^+^*e*^−^, *A*_φ_ = (−0.6 ± 3.1) × 10^−2^ [72], from the KLOE experiment is compatible with zero. At BESIII, taking into account the measured branching fraction for η^′^ → π^+^π^−^*e*^+^*e*^−^, (2.11 ± 0.12 ± 0.15) × 10^−3^ [73], about 2 × 10^4^ events could be used to explore the *CP* violation using the full data sample of 10 billion *J*/ψ events. More recently, the η^′^ → π^+^π^−^μ^+^μ^−^ decay was observed for the first time in the BESIII experiment [74].

Experimentally, η/η^′^ → *l*^+^*l*^−^π^0^ decays could be used to test charge-conjugation invariance. In the SM, this process can proceed via a two-virtual-photon exchange whereas one-photon-exchange violates *C* parity. Within the framework of the VMD model, the most recent predictions [75] for the branching fraction are of the order of 10^−9^ for η → *l*^+^*l*^−^π^0^ and 10^−10^ for η^′^ → *l*^+^*l*^−^π^0^(η). Thus, a significant enhancement of the branching fractions exceeding the two-photon model may be indicative of *C* violation. With the available 10 billion *J*/ψ events, further improvement for these rare decays will be achieved.

## LIGHT QUARK VECTOR MESONS IN *e*^+^*e*^−^ ANNIHILATION

Information on light vector meson decays has been obtained from *e*^+^*e*^−^ annihilations in, e.g. the KLOE, SND, CMD-2, BaBar and Belle experiments (see [76] for a review), where the vector mesons are observed as the peaks in the total cross section for the specific final states when the *e*^+^*e*^−^ center of mass energy is varied by tuning the beam energy or by the initial state radiation (ISR) process. With energy scan data in the 2.0–3.08 GeV range, BESIII can perform direct searches for light vector mesons, especially the poorly studied vector strangeonium states.

**Figure 5.**
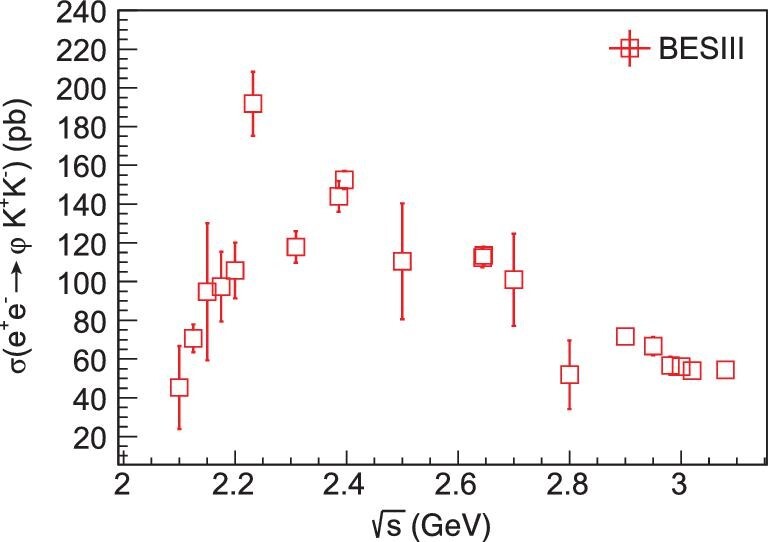
The measured Born cross section of *e*^+^*e*^−^ → φ*K*^+^*K*^−^ [83].

The φ(2170), previously referred to as the Y(2175), has been established in the BaBar [77] and BES [78] experiments, but its measured mass and width remain controversial. There have been a number of different interpretations for φ(2170), such as a conventional $s\bar{s}$ state, a QCD hybrid, a tetraquark state, a $\Lambda \bar{\Lambda }$ bound state or a $\phi K\bar{K}$ resonance state. The situation will not be clarified without further experimental data. At BESIII, the line shapes of the cross sections for a number of measured channels, including *e*^+^*e*^−^ → *K*^+^*K*^−^ [79], *e*^+^*e*^−^ → *K*^+^*K*^−^π^0^π^0^ [80] and *e*^+^*e*^−^ → φη^′^ [81], were measured and a clear structure around 2.2 GeV was evident in each of them. The measured widths and masses are consistent with those from *J*/ψ → φπ^+^π^−^η [82], as summarized in Table. 1. Of interest is the process of *e*^+^*e*^−^ → *K*^+^*K*^−^*K*^+^*K*^−^ [83], and its dominant submode *e*^+^*e*^−^ → φ*K*^+^*K*^−^. The line shape for the latter is shown in Fig. 5. In both cases, a very narrow enhancement at $\sqrt{s}= 2.232$ GeV is observed, which is very close to the $e^{+}e^{-} \rightarrow \Lambda \overline{\Lambda }$ production threshold.

**Table 1.** Summary of the mass and width of φ(2170) obtained from the BESIII experiment.

Process	Mass (MeV/c^2^)	Width (MeV)
*e* ^+^ *e* ^−^ → *K*^+^*K*^−^ [79]	2239.2 ± 7.1 ± 11.3	139.8 ± 12.3 ± 20.6
*e* ^+^ *e* ^−^ → *K*^+^*K*^−^π^0^π^0^ [80]	2126.5 ± 16.8 ± 12.4	106.9 ± 32.1 ± 28.1
*e* ^+^ *e* ^−^ → φη^′^ [81]	2177.5 ± 4.8 ± 19.5	149.0 ± 15.6 ± 8.9
*J*/ψ → φπ^+^π^−^η [82]	2200 ± 6 ± 5	104 ± 15 ± 15

Another interesting possible strangeonium candidate is the *X*(1750) observed in the photoproduction process [84], which was originally interpreted as the photoproduction mode of the φ(1680). However, the recent simultaneous observation of the φ(1680) and *X*(1750) in ψ(2*S*) → *K*^+^*K*^−^η decays [85] indicates that the *X*(1750) is distinct from the φ(1680) and possibly a strangeonium state.

The above examples demonstrate that BESIII is a powerful instrument for investigating the light vector mesons. At present, more studies, such as *e*^+^*e*^−^ → φπ^+^π^−^, *e*^+^*e*^−^ → φη and *J*/ψ → *K*^+^*K*^−^η, are ongoing to provide a deeper understanding of the nature of the φ(2170) and *X*(1750), and to search for new strangeonium states.

## SUMMARY AND PROSPECTS

The light meson decays, as described above, provide a unique opportunity to investigate many aspects of particle physics at low energy, with the advantages of high production rates and excellent performance of the detector. In addition to improved accuracy on many of the measured properties of well-known light meson decays, a series of first observations, such as new decay modes of η^′^, *a*_0_(980) − *f*_0_(980) mixing as well as possibly new strangeonium states, were reported. These significant advances demonstrate that BESIII is playing a leading role in the study of light meson decays.

Despite this impressive progress, many light meson decays are still unobserved and need to be explored. At BESIII, 10^10^*J*/ψ events data are now available. This is 8 times larger than the subdata sample used in the present publications and offers great additional opportunities for research in light meson decays, especially for pseudoscalar and vector mesons, with unprecedented precision. Moreover, BESIII expects to take an additional 20 fb^−1^ of data at 3.773 GeV, which will support investigations of the light meson physics with different ISR and two-photon production techniques, such as the production of new vector mesons and measurements of the two-photon width of the light scalar mesons. In addition, different experimental techniques will give access to previously unexplored regions of the electromagnetic transition form factors, allowing a quantitative connection between the timelike and the spacelike regions.

In general, together with the other high-precision experiments, such as KLOE-2, A2, GlueX and BelleII, these very abundant and clean event samples that are accumulated at BESIII will bring the study of light meson decays into a precision era, and will definitely play an important role in the development of chiral effective field theory and lattice QCD, and make significant contributions to the understanding of hadron physics in the non-perturbative regime.
